# A GWAS-identified susceptibility locus on chromosome 11q13.3 and its putative molecular target for prediction of postoperative prognosis of human renal cell carcinoma

**DOI:** 10.3892/ol.2013.1422

**Published:** 2013-06-25

**Authors:** TONG SU, YIFANG HAN, YONGWEI YU, XIAOJIE TAN, XIAOPAN LI, JIANGUO HOU, YAN DU, JIAN SHEN, GUOPING WANG, LIYE MA, SHUANG JIANG, HONGWEI ZHANG, GUANGWEN CAO

**Affiliations:** 1 Department of Epidemiology, Second Military Medical University;; 2 Departments of Pathology, Changhai Hospital, Second Military Medical University, Shanghai 200433, P.R. China; 3 Urology, Changhai Hospital, Second Military Medical University, Shanghai 200433, P.R. China; 4 General Surgery, Changhai Hospital, Second Military Medical University, Shanghai 200433, P.R. China; 5 Physical Examination Center, Changhai Hospital, Second Military Medical University, Shanghai 200433, P.R. China

**Keywords:** single nucleotide polymorphism, renal cell carcinoma, risk, prognosis, cyclin D1

## Abstract

Genome-wide association studies have been used to identify single nucleotide polymorphisms (SNPs) associated with renal cell carcinoma (RCC) in European individuals. The current study aimed to evaluate the correlation between significant SNPs identified in European individuals and the occurrence and postoperative prognosis of RCC in Chinese individuals. A total of 400 cases and 806 controls were involved in the current study. rs4765623, rs7105934, rs7579899 and rs1867785 were genotyped using qPCR, and the expression of cyclin D1 in renal tissue and RCCs was determined via western blotting and immunohistochemistry. The correlation between the SNPs/cyclin D1 expression and overall survival was evaluated using multivariate Cox regression analyses. Of the four SNPs, only rs7105934 was found to significantly correlate with RCC risk in Chinese individuals. The rs7105934 GA + AA genotype was correlated with a reduced risk of RCC with an odds ratio of 0.64 (95% confidence interval [CI], 0.43–0.96), following adjustment for age. This genotype was found to independently predict an improved postoperative prognosis in the multivariate analysis, with a hazard ratio (HR) of 0.12 (95% CI, 0.02–0.93). Expression of cyclin D1, a putative regulated protein of rs7105934, did not vary in adjacent renal tissue and tumors when compared with that of various rs7105934 genotypes. However, cyclin D1 expression in RCCs inversely correlated with advanced tumor stage, and moderate to high expression of cyclin D1 in RCCs independently predicted improved postoperative prognosis, with an HR of 0.13 (95% CI, 0.02–0.96). Observations of the present study indicate that the rs7105934 A allele is associated with reduced risk and improved postoperative prognosis of RCC; however, this effect is unlikely to be caused by cyclin D1 expression.

## Introduction

Renal cell carcinoma (RCC) accounts for 80–90% of cases of kidney cancer. The incidence rate of RCC is generally higher in more developed countries than in less developed countries (1). The rate of RCC is 2-fold higher in males than in females and increases with age. RCC is a pathogenically heterogeneous disease and clear cell RCC (ccRCC) accounts for 70–80% of cases of kidney cancer. Epidemiological studies have identified that hypertension, obesity, cigarette smoking and exposure to trichloroethylene are environmental risk factors of RCC; however, the impact of these factors may vary among populations (2–4). Environmental factors and their interaction with genetic factors are hypothesized to affect the risk of RCC (5,6). Single nucleotide polymorphisms (SNPs) in cancer growth-related cytokines, including vascular endothelial growth factor, Dickkopf-3 in the Wnt pathway and blood pressure genes, are associated with RCC risk (7–9). SNPs of metabolic enzymes, including cytochrome P450 mono-oxygenases, N-acetyltransferase and glutathione S-transferase, are associated with RCC risk in Caucasians; although, these correlations have not been replicated in Chinese individuals (4,10), indicating that the association between genetic predisposition and RCC risk alters among various populations. However, a number of studies using candidate-gene approaches have not yielded a conclusive result (4–10).

Surgical resection remains the curative therapy for RCC patients; although, patients treated surgically have 5- and 10-year relative survival rates of 72 and 63%, respectively (11). Previous studies have shown that specific clinical and molecular factors have predictive values for postoperative prognosis: Advanced tumor stages; circulating molecules, including C-reactive protein and erythrocyte polyamines; molecules in the tumor, including L1 cell adhesion molecules; and chromosomal variations, including 10q and 13q deletions, and D9S168 micro-satellite alterations. These factors are capable of predicting a poor postoperative prognosis for RCC (12–16). Genetic predispositions, including the Dickkopf-2 rs17037102 polymorphism, interleukin-4 haplotype -589T-33T, *MDM2* rs2279744 polymorphism and miRNA-related genetic polymorphisms are closely associated with RCC clinical outcome (8,17–19).

A previous genome-wide association study (GWAS) in RCC cases and controls of European background revealed that two loci mapped to *EPAS1,* encoding hypoxia inducible factor (HIF)-2α, on 2p21 (rs11894252 and rs7579899), a locus on 11q13.3 (rs7105934) and a locus mapped to *SCARB1,* encoding the scavenger receptor class B, member 1, on 12q24.31 (rs4765623) were significantly associated with RCC susceptibility (20). rs7105934 at 11q13.3 was detected to modulate the binding and function of HIF-2α at a transcriptional enhancer of *CCND1* (encoding cyclin D1) (21). In the present study, the correlations between SNPs identified in the present GWAS and RCC susceptibility were validated, and the role of SNPs in the postoperative prognosis of RCC was investigated in a Chinese population.

## Materials and methods

### Study subjects

A total of 400 pathologically confirmed, sporadic RCC patients diagnosed between November 1998 and November 2011 at the Department of Urology (Changhai Hospital, Second Military Medical University, Shanghai, China) were involved in the present study. In addition, 806 controls that received comprehensive physical examinations, including type-B ultrasonic and blood tests, and were confirmed to be healthy at the Physical Examination Center of Changhai Hospital between 2006 and 2011, were also involved. Individuals who did not have notable metastasis at the time of surgery and did not receive adjuvant therapy after surgery were followed up. Follow-up was initiated 6 months after surgery, performed by telephone or interviews in person at the Outpatient Department every 3 months, in accordance with standard epidemiological procedures. The median follow-up period was 34.0 months (range, 3.0–90.9 months). All participants were of Chinese ethnic origin, and the study protocol conformed to the ethical guidelines of the 1975 Declaration of Helsinki and was approved by the Institutional Review Board of the Second Military Medical University (Shanghai, China). All participants provided written informed consent.

### Genotyping of genetic polymorphisms

Genomic DNA was extracted from blood samples using QIAquick PCR purification kits (Qiagen, Hilden, Germany). rs4765623, rs7105934, rs7579899 and rs1867785 (highly correlated with rs11894252) were genotyped using fluorescent-probe qPCR in a LightCycler^™^ 480 (Roche Diagnostics, Mannheim, Germany). Primers and probes (TaqMan or Minor Groove Binder) were designed and synthesized by GeneCore BioTechnologies Co., Ltd. (Shanghai, China), and the sequences of the primers and probes are shown in Table I. Each reaction mixture contained 0.2 *μ*mol/l primers and probes, as well as 0.1–0.5 *μ*g purified templates in the Premix Ex Taq^™^ reaction system (Takara Bio, Inc., Shiga, Japan). Three duplicative samples were run with a template-free control.

### Western blotting

Protein was extracted from the adjacent renal tissue of fresh RCC specimens and quantified using cell lysis buffer and Enhanced BCA Protein Assay kits (Beyotime Institute of Biotechnology, Jiangsu, China). Extracted proteins were resolved by routine PAGE gels and transferred onto polyvinylidene difluoride membranes. The membranes were incubated overnight with rabbit monoclonal antibodies against cyclin D1 (1:1,000; Epitomics Inc., Burlingame, CA, USA) and β-ctin (1:1,000; Cell Signaling Technology, Inc., Danvers, MA, USA), washed and incubated with HRP-conjugated anti-rabbit or anti-mouse IgG (Cell Signaling Technology, Inc.) for 2 h. Peroxidase activity was developed by ECL detection reagent (GE Healthcare, Amersham, UK) according to the manufacturer’s instructions. The signal intensity of each band was quantified using Genetools software, version 4.02 (Syngene, Cambridge, UK). The relative intensity of each sample was calculated as the intensity of the cyclin D1 band (raw value-background noise)/the intensity of the β-actin band (raw value-background noise).

### Immunohistochemistry

Antibodies against cyclin D1 (1:100) were used for immunohistochemistry of RCC specimens in accordance with methods previously described for western blotting (22). Sections were independently assessed by four researchers who were blinded to the clinical information. Immunostaining intensity was scored as follows: Negative (−), <5% positive cancer cells; weak (+), 5–20% positive; moderate (++), 20–60% positive; and strong (+++), >60% positive. There was >95% agreement among the researchers and disagreements were resolved by consensus. A total of 90 paraffin-embedded samples were used in immunohistochemical assay.

### Statistical analysis

Differences in categorical variables were evaluated using the χ^2^ test. Hardy-Weinberg equilibrium (HWE) was examined online (http://ihg.gsf.de/ihg/snps.html) and linkage disequilibrium (LD) analyses for the SNPs involved in the current study and those identified in previous GWAS (23,24) were performed using online Haploview 4.2 software (http://hapmap.ncbi.nlm.nih.gov). Differences in the relative intensities of the western blotting bands among the samples of various genotypes were analyzed using the Student’s t-test. Unconditional logistic regression analysis was used to obtain an odds ratio (OR) for each SNP with RCC and a 95% confidence interval (CI). Overall survival was analyzed using the Kaplan-Meier method and the log-rank test was used to compare survival curves. Forward stepwise multivariate Cox regression analysis (P_entry_=0.05 and P_removal_=0.10) was performed to determine the factors contributing independently to RCC prognosis. The correlation between immunostaining scores for cyclin D1 and RCC stage and the rs7105934 genotypes was determined by Spearman’s rank correlation analysis, respectively. All statistical tests were two-sided and conducted using SPSS for Windows, version 16.0 (SPSS, Inc., Chicago, IL, USA). P<0.05 was considered to indicate a statistically significant difference.

## Results

### Correlation between the four SNPs and RCC risk

Table II shows the characteristics of the RCC patients and healthy controls from the present study. The gender distribution was not significantly different between the RCC patients and healthy controls; however, the healthy controls were significantly older than the RCC patients (median ± standard deviation; 60.1±12.8 vs. 57.1±12.8 years; P<0.001). The genotyping call rate was 100% for each SNP, and in the healthy controls, rs4765623, rs7105934, rs7579899 and rs1867785 conformed to HWE (P=0.481, 0.891, 0.352 and 0.373, respectively). LD analysis indicated that rs7579899 was markedly correlated with rs1867785 (r^2^=0.988 in this study population), whereas rs1867785 was not in LD with rs12617313 or rs9679290 (r^2^<0.05 in the HapMap Chinese population), the SNPs identified on chromosome 2p21 by a previous GWAS (24). Table III shows the correlation between the SNPs and RCC risk. Of the four SNPs, rs7105934 only was found to significantly correlate with RCC risk. The dominant model or A allele at rs7105934 was significantly correlated with a reduced risk of RCC following adjustments for age and gender, with a power of 77.4%. As ccRCC is the major histological type, the correlation between the four SNPs and ccRCC risk was examined, and rs7105934 only was found to correlate with the risk of ccRCC. Compared with the GG genotype, the GA + AA genotype of rs7105934 was significantly correlated with a reduced ccRCC risk with an OR of 0.60 (95% CI, 0.40–0.92; P= 0.018), following adjustments for age and gender. By contrast, the A allele of rs7105934 was inversely associated with ccRCC risk following adjustments (OR, 0.62; 95% CI, 0.41–0.92; P=0.018).

### Correlation between SNPs and RCC prognosis

**Figure 1.**
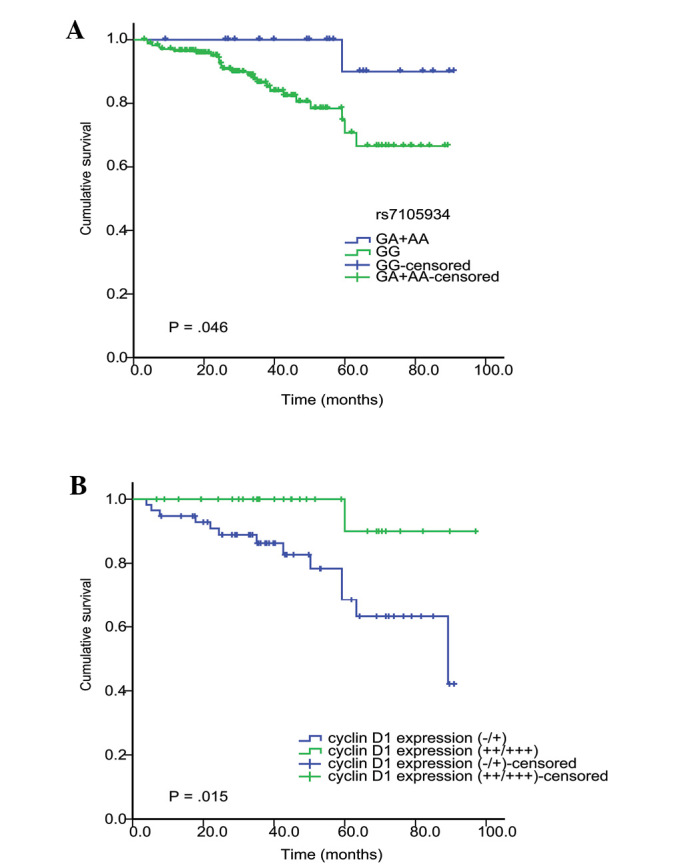
Correlation between rs7105934 or cyclin D1 protein expression in tumor tissues and overall survival of RCC patients following curative nephrectomy (Kaplan-Meier analysis and log-rank test). (A) rs7105934 and overall survival (n=194). (B) Cyclin D1 protein expression in tumor tissues and overall survival (n=90). −, negative (<5% positive cancer cells); +, weak (5–20% positive); ++, moderate (20–60% positive); +++, strong (>60% positive); RCC, renal cell carcinoma.

Of the RCC patients, 194/400 completed the follow-up and were included in the survival analysis. It was identified that rs7105934 GA + AA genotype was significantly correlated with an improved postoperative prognosis of RCC (P=0.046; Kaplan-Meier and log-rank test; Fig. 1A). Table IV presents the results of stepwise multivariate Cox regression analysis, indicating that patients with the rs7105934 GA + AA genotype have a decreased risk of mortality (HR, 0.12; 95% CI, 0.016–0.93; P=0.042). In addition, advanced tumor stage was also found to independently predict poor survival rates in RCC.

### Correlation between rs7105934 genotypes and cyclin D1 expression

**Figure 2.**
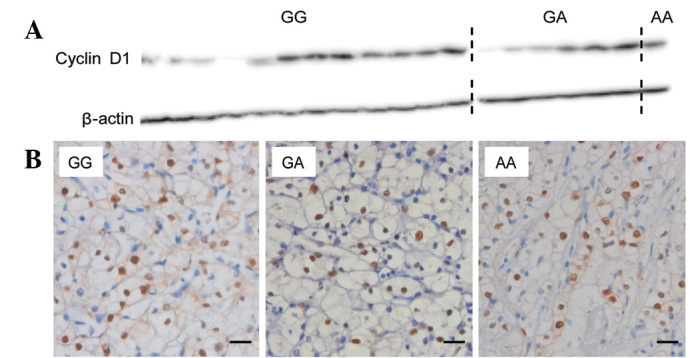
Correlation between rs7105934 genotypes and the expression of cyclin D1 in adjacent renal and RCC tissues. (A) Western blotting to determine cyclin D1 expression in adjacent renal tissue. (B) Immunohistochemistry for the analysis of cyclin D1 expression in RCC tissue. Bar indicates 20 *μ*m and brown indicates positive immunostaining. RCC, renal cell carcinoma.

Expression of cyclin D1 protein in the adjacent renal tissue of fresh RCC specimens was examined using western blotting. No significant differences were identified in the relative intensity of cyclin D1 protein between patients with the GG genotype and those with the GA + AA genotype of rs7105934 (P=0.514; Fig. 2A). Cyclin D1 expression in the tumors was examined using immunohistochemistry (Fig. 2B). Immunostaining intensities of cyclin D1 in the cancer cells of the RCC patient specimens did not correlate significantly among the three genotype groups of rs7105934 (P=0.844) or the GG and GA + AA genotypes of rs7105934 (P=0.884). However, immunostaining intensities of cyclin D1 in the cancer cells was inversely correlated with advanced tumor stage (P=0.046; Spearman’s correlation coefficient, −0.211; Table V).

### Correlation between cyclin D1 expression in cancer cells and RCC prognosis

The association between cyclin D1 expression in cancer cells and RCC prognosis was first evaluated using Kaplan-Meier analysis. Moderate to high expression (++/+++) of cyclin D1 in cancer cells predicted an improved postoperative prognosis of RCC when compared with that of negative or weak expression (−/+) (P=0.015; Kaplan-Meier analysis and the log-rank test; Fig. 1B). Multivariate Cox regression analysis indicated that moderate and high expression (++/+++) of cyclin D1 in cancer cells independently predicted an improved postoperative prognosis (HR, 0.13; 95% CI, 0.02–0.96; P=0.045).

## Discussion

In the present study, the correlation between four SNPs identified in a previous GWAS study of a European population and the development and prognosis of RCC in a Chinese population was investigated. In the current study, the healthy controls and RCC patients were matched by gender, but the healthy controls were significantly older than the RCC patients. This age contrast may have prevented misclassification bias, as RCC incidence increases with age (2). The current study identified that, of the four SNPs validated, only rs7105934 significantly correlated with the risk of RCC, particularly ccRCC, in the Chinese population. An additional study investigating the association between rs7579899, rs7105934 and rs4765623 and RCC risk in an additional group of Chinese individuals has been reported (25). Similar to the results of the current study, the study identified that only rs7105934 of the genotyped SNPs correlated with a reduced risk of RCC. Therefore, genetic predisposition of RCC appears to differ between the two ethnic groups and rs7105934 may confer genetic susceptibility to RCC in a number of populations.

An important observation of the present study was that the rs7105934 GA + AA genotype is an independent factor for improved RCC prognosis, with an HR of 0.12 (95% CI, 0.02–0.93), in contrast to advanced tumor stage. The genetic risk factor of RCC development may also modulate RCC outcome. rs7105934 is situated at a transcriptional enhancer ∼220-kb telomeric to the cyclin D1 encoding gene, *CCND1* (20). SNPs 5-kb centromeric to rs7105934 modulate the binding and function of HIF-2α at the enhancer for *CCND1.* The minor (RCC-protective) allele at 11q13.3 disrupts HIF binding, DNA accessibility and interaction with the transcriptional apparatus at the *CCND1* enhancer, and alters the allelic balance of *CCND1* gene expression (21). The rs7105934 GA genotype appears to be associated with lower levels of *CCND1* mRNA in adjacent renal tissue when compared with that of the GG genotype (25). Therefore, the correlation between the GA + AA genotype and an improved prognosis of RCC may be caused by low expression of cyclin D1 in tumors. Of note, the present study identified that moderate to high expression of cyclin D1 is an independent predictor of an improved postoperative prognosis in RCC. No significant differences were identified in cyclin D1 protein expression between specimens with the GG genotype and those with the GA + AA genotype in adjacent renal tissue and in the tumor tissue. In addition, the intensity of cyclin D1 immunostaining did not correlate with the rs7105934 genotype, and these results were not consistent with the hypothesized involvement of rs7105934 genotypes in cyclin D1 expression in RCCs. Thus, cyclin D1 and the rs7105934 SNP may both be significant in the carcinogenesis and postoperative prognosis of RCC, but function independently. Further studies are required to validate and investigate the correlation between rs7105934 genotypes and the expression of cyclin D1 and other candidate target proteins in RCCs.

In the present study, cyclin D1 expression in RCC cells was confirmed to inversely correlate with advanced stages of RCC and represent an independent predictor of improved prognosis of RCC. The results of the current study are similar to those of previous studies reporting that high cyclin D1 expression significantly correlates with an improved prognosis, while low expression of cyclin D1 correlates with a poor prognosis of ccRCC (26–28). The expression of cyclin D1 is markedly correlated with that of p27 in ccRCC; low p27 expression independently predicts a poor prognosis of RCC, whilst low cyclin D1 expression appears to shorten the survival rate of RCC patients (29). These differences may reflect variations in the cyclin D1-related cell biology of various cancer types. Cyclin D1 is an oncoprotein that activates the G_1_-S transition of the cell cycle and functions as a downstream effector of β-catenin signaling. Downregulation of β-catenin expression may contribute to the malignant character of RCC and result in tumor progression (30). Cyclin D1 is upregulated in primary ccRCCs, which may contribute to cell proliferation in primary RCCs, and its importance may diminish at later stages of RCC progression due to specific complex mechanisms, including epithelial-mesenchymal transition.

Several limitations of the present study must be noted: i) follow-up information was only available for 194 study subjects and the majority of individuals without follow-up information were from other provinces of China, which made regular contact difficult; ii) patients were excluded in cases where postoperative therapy was administered, including interferon-α and interleukin-2 treatments, during the follow-up period; iii) epidemiological studies of specific risk factors, including smoking and alcohol consumption, were incomplete and thus not included in the analysis, resulting in loss of data; and iv) SNPs were validated from only one GWAS study (20) and novel RCC-related SNPs on 12p11.23 and 2p21 (23,24) require validation, as these SNPs are not in LD with the four SNPs validated for the present study.

Overall, the present study showed that, of the four RCC-related SNPs identified in a European population, only rs7105934 correlated with RCC risk in a Chinese population. The GA + AA genotype was significantly correlated with reduced risk and an improved postoperative prognosis of RCC. Therefore, rs7105934 may confer genetic susceptibility to RCC in extended populations. No significant differences were identified in the levels of cyclin D1 expression in adjacent renal tissue and RCC cells between patients with the rs7105934 GG genotype and those with the GA + AA genotype. Cyclin D1 expression in RCC cells was inversely correlated with advanced stages of RCC. In addition, moderate to high expression of cyclin D1 was an independent predictor of improved postoperative prognosis of RCC. However, the results do not support the hypothesis that rs7105934 genotypes are involved in the expression of cyclin D1 in the kidneys. Cyclin D1 and the rs7105934 SNP may represent significant factors in RCC, however, their effects appear to be independent. Although validation of these observations is required in independent populations, the current study highlights a novel genetic marker that is capable of predicting the postoperative prognosis of RCC.

## Figures and Tables

**Table I.** Primers and probes for genotyping the four single nucleotide polymorphisms.

	Variation	Primers 5′-3′	Probes
rs7105934	G/A	GAGGAATGATGAACAAACTGTGGTA	FAM-CCAAAATGCATCGTGCTAAGAAGCC-TAMRA
		CAGAACATCACATAAATGGAATCATACA	HEX-TCCAAAATGCATCATGCTAAGAAGCC-TAMRA
rs1867785	A/G	GGACTTCTCTCTCCCTTCACCCT	FAM-AAATTAGCTTCGTTGACCTCAGCCAGC-TAMRA
		TCCTGTGTTTCCAAGAGTTCTCAGA	HEX-ATTAGCTTCGTCGACCTCAGCCAGC-TAMRA
rs7579899	A/G	ACACAGCCAAATCCAAGTCAGA	FAM-ACACCCTGTACAAAGCACTGCGACC-TAMRA
		TGACCAAACACTAGGAAAGGAGAAG	HEX-ACACCCTGTACAGAGCACTGCGACC-TAMRA
rs4765623	C/T	GGTCTCGCGCATGTGTCA	FAM- AGTACAGCCACCTCGGAGAGCCACT-TAMRA
		CCAGATGCGTTCAGCAGTTC	HEX- AGTACAGCCACCTTGGAGAGCCACTG-TAMRA

**Table II.** Demographic and clinicopathological characteristics of the study subjects.

Characteristics	Cases, n (%)	Controls, n (%)	P-value
Age, years			
≤40	31 (7.8)	36 (4.5)	0.008
40–60	210 (52.5)	380 (47.1)	
60–80	147 (36.8)	352 (43.7)	
>80	12 (3.0)	38 (4.7)	
Gender			
Male	274 (68.5)	548 (68.0)	0.858
Female	126 (31.5)	258 (32.0)	
Histology			
Clear cell	373 (93.3)	-	
Papillary	12 (3.0)	-	
Chromophobe	8 (2.0)	-	
Unclassified	7 (1.8)	-	
AJCC (2002) stage			
I	324 (81.0)	-	
II	33 (8.3)	-	
III	43 (10.8)	-	

AJCC, American Joint Committee on Cancer.

**Table III.** Correlation between the four SNPs and risk of RCC following adjustment for age and gender.

Genotype	Cases, n (%)	Controls, n (%)	OR (95% CI)	P-value^a^
rs7105934				
GG	364 (91.0)	700 (86.8)	1.00 (reference)	
GA	35 (8.8)	102 (12.7)	0.65 (0.43–0.97)	0.036
AA	1 (0.3)	4 (0.5)	0.47 (0.05–4.25)	0.500
GG	364 (91.0)	700 (86.8)	1.00 (reference)	
GA + AA	36 (9.0)	106 (13.2)	0.64 (0.43–0.96)	0.029
G allele	763 (95.4)	1502 (93.2)	1.00 (reference)	
A allele	37 (4.6)	110 (6.8)	0.65 (0.44–0.95)	0.028
rs1867785				
AA	288 (72.0)	561 (69.6)	1.00 (reference)	
AG	105 (26.3)	227 (28.2)	0.90 (0.69–1.19)	0.472
GG	7 (1.8)	18 (2.2)	0.76 (0.31–1.86)	0.552
AA	288 (72.0)	561 (69.6)	1.00 (reference)	
AG + GG	112 (28.0)	245 (30.4)	0.89 (0.69–1.17)	0.411
A allele	681 (85.1)	1349 (83.7)	1.00 (reference)	
G allele	119 (14.9)	263 (16.3)	0.90 (0.71–1.14)	0.381
rs7579899				
AA	287 (71.8)	560 (69.5)	1.00 (reference)	
AG	106 (26.5)	228 (28.3)	0.91 (0.69–1.19)	0.495
GG	7 (1.8)	18 (2.2)	0.76 (0.31–1.86)	0.554
AA	287 (71.8)	560 (69.5)	1.00 (reference)	
AG + GG	113 (28.3)	246 (30.5)	0.90 (0.69–1.17)	0.432
A allele	680 (85.0)	1348 (83.6)	1.00 (reference)	
G allele	120 (15.0)	264 (16.4)	0.90 (0.71–1.14)	0.399
rs4765623				
CC	136 (34.0)	268 (33.3)	1.00 (reference)	
CT	188 (47.0)	385 (47.8)	0.97 (0.74–1.28)	0.845
TT	76 (19.0)	153 (19.0)	0.97 (0.68–1.37)	0.844
CC	136 (34.0)	268 (33.3)	1.00 (reference)	
CT + TT	264 (66.0)	538 (66.7)	0.97 (0.75–1.25)	0.822
C allele	460 (57.5)	921 (57.1)	1.00 (reference)	
T allele	340 (42.5)	691 (42.9)	0.98 (0.83–1.17)	0.826

a P-values were compared with the corresponding reference. SNPs, single nucleotide polymorphisms; RCC, renal cell carcinoma; OR, odds ratio.

**Table IV.** Factors independently associated with overall survival of RCC patients in stepwise multivariate Cox regression analysis.

	Cases evaluated, n	Cases of RCC mortality, n	HR (95% CI)	P-value
rs7105934				
GG	172	25	1.00 (reference)	
GA + AA	22	1	0.12 (0.016–0.93)	0.042
AJCC (2002) stage				
I	147	7	1.00 (reference)	
II	12	3	5.79 (1.47–22.82)	0.012
III	35	16	15.72 (6.36–38.84)	<0.001

RCC, renal cell carcinoma; HR, hazard ratio; AJCC, American Joint Committee on Cancer.

**Table V.** Correlation between rs7105934 genotypes or AJCC stage and cyclin D1 expression in ccRCC specimens.

	Cyclin D1 expression			P-value	r_s_^a^
	−	+	++/+++		
rs7105934					
GG	24	25	27	0.844	0.021
GA	5	3	5		
AA	0	0	1		
GG	24	25	27	0.884	0.016
GA + AA	5	3	6		
AJCC (2002) stage					
I	16	15	26	0.046	−0.211
II	3	3	2		
III	10	10	5		

a Spearman’s correlation coefficient. AJCC, American Joint Committee on Cancer; −, negative (<5% positive cancer cells); +, weak (5–20% positive); ++, moderate (20–60% positive); +++, strong (>60% positive); ccRCC, clear cell renal cell carcinoma.
